# MicroRNA Machinery Genes as Novel Biomarkers for Cancer

**DOI:** 10.3389/fonc.2014.00113

**Published:** 2014-05-19

**Authors:** Jing-Tao Huang, Jin Wang, Vibhuti Srivastava, Subrata Sen, Song-Mei Liu

**Affiliations:** ^1^Center for Gene Diagnosis, Zhongnan Hospital of Wuhan University, Wuhan, China; ^2^Department of Translational Molecular Pathology, The University of Texas MD Anderson Cancer Center, Houston, TX, USA

**Keywords:** miRNA, machinery genes, alterations, biomarker, cancer

## Abstract

MicroRNAs (miRNAs) directly and indirectly affect tumorigenesis. To be able to perform their myriad roles, miRNA machinery genes, such as *Drosha*, *DGCR8*, *Dicer1*, *XPO5*, *TRBP*, and *AGO2*, must generate precise miRNAs. These genes have specific expression patterns, protein-binding partners, and biochemical capabilities in different cancers. Our preliminary analysis of data from The Cancer Genome Atlas consortium on multiple types of cancer revealed significant alterations in these miRNA machinery genes. Here, we review their biological structures and functions with an eye toward understanding how they could serve as cancer biomarkers.

## Introduction

MicroRNAs (miRNAs) are non-coding RNAs ~22 nt long that bind to target mRNAs, resulting in mRNA degradation or inhibition of mRNA expression (1, 2), and play a key role in post-transcriptional gene regulation in up to 30–60% of all human genes (3). miRNA targets mRNA by specific base-pairing interactions between the seed region of the miRNA and the 5′-untranslated regions of the mRNA (4–6). miRNAs can be grouped into families on the basis of their seed sequences, and members of one family usually effect the same mRNAs. A small number of miRNAs outside the seed sequences have also been reported (7, 8).

MicroRNAs can be produced from long RNA transcripts. Primary miRNAs (pri-miRNAs), which are 1–2 kb long and contain one or more 70-nt hairpin precursor miRNAs (pre-miRNAs), are excised to pre-miRNAs by ribonuclease III (RNase III) and DiGeorge critical region 8 (*DGCR8*) in the cell nucleus (9–11). The *Drosha–DGCR8* complex, known as a microprocessor, is essential for miRNA maturation. *Drosha*, as the catalytic subunit, has been shown to cleave pri-miRNA-like hairpins harbored within the 5′-untranslated region of the mRNA encoding the *DGCR8* protein (12, 13). *Drosha* is a member of the RNase III family and can convert pri-miRNAs into pre-miRNAs (11), which are exported from the nucleus into the cytoplasm by an exportin-5 (*XPO5*)/*Ran–GTP* complex (14–16). In the cytoplasm, the endoribonuclease *Dicer* complex catalyzes these pre-RNAs to form miRNAs (17). The mature miRNAs are loaded into an argonaute 2 (*AGO2*) protein, which associates with a TAR RNA-binding protein (*TRBP*) and forms an RNA-induced silencing complex (RISC) (18, 19), which plays a crucial role in the repression or degradation of mRNAs.

## miRNA Machinery Genes

The miRNA machinery genes include *Drosha*, *DGCR8*, *Dicer1*, *XPO5*, *TRBP*, and *AGO2*, which synthesize proteins to regulate the processing of miRNAs and influence different fields *in vivo*. *Drosha*, a nuclear RNase III enzyme, has two RNase III catalytic sites with a double-stranded RNA-binding domain (dsRBD) at the C terminus and a proline-rich domain and arginine/serine-rich domains at the N terminus (11). *Drosha* recognizes the stem-loop structure and cleaves both arms of the stem-loop through the tandem RNase III domains. The RNase III family of enzymes, which are found in all eubacteria and eukaryotes (20), is divided into three classes based on their structure. Of these classes, *Drosha* class II and *Dicer* class III have crucial effects on miRNA processing. The long pri-miRNA, which is typically generated by RNA polymerase II, contains a short stem-loop structure (11). *DGCR8* can stabilize the *Drosha* protein through protein–protein interaction (12) and is an essential miRNA processing factor that includes an N-terminal region for nuclear localization, a heme-binding domain, two dsRBDs, and a C-terminal tail (21, 22). *DGCR8* binds to the base of the long primary transcript pri-miRNA hairpin, positioning *Drosha* to cleave the pri-miRNA stem at a distance of 11 base pairs from the junction between the double-stranded RNA (dsRNA) stem and the flanking single-stranded RNA regions (23).

*XPO5* is a nuclear receptor that transports pre-miRNA from the nucleus to the cytoplasm (24, 25). Once in the cytoplasm, pre-miRNA is cleaved by *Dicer* in complex with another dsRNA-binding protein, *TRBP* (19, 25, 26). As a key protein in the cleaving process of pri-miRNA, *Dicer* has two RNase III domains, a less-conserved *ATPase/DExD* helicase domain and a Piwi–Argonaute–Zwille (*PAZ*) domain (27, 28). The key regions for miRNA maturation, these domains have different effects. The RNase IIIA domain of *Dicer1* is essential for generating small RNAs embedded in the 3′ stem of exogenous hairpin-like RNAs (29). Inactivation of this domain results in complete loss of *3p*-derived mature miRNAs but only partial reduction in *5p*-derived mature miRNAs (30). In contrast, inactivation of the RNase IIIB domain by mutation of D1709 results in complete loss of *5p*-derived mature miRNAs but only partial reduction in *3p*-derived mature miRNAs. Mutation of the *PAZ* domain in *Dicer* results in global reduction of miRNA processing (30).

Argonaute proteins are core components of RISCs and are highly conserved between species. Many organisms encode multiple members of this protein family, which have essential roles in RNA-mediated gene silencing (31). *AGO2* protein contains four major domains, N-terminal, *PAZ* domain, *MID* domain, and *PIWI* domain (32, 33), as well as two structured linker domains, L1 and L2 (34). The *PAZ* domain, like *Dicer*, binds to the 3′ end of guide RNA (35). The *MID* domain of the eukaryotic *AGO* protein *QDE-2* adopts a Rossmann-like fold and recognizes the 50-nt terminal of a guide RNA in a manner similar to its prokaryotic counterparts, for which the 50-nt-binding site shares common residues with a second, adjacent ligand-binding site (36).

TAR RNA-binding protein is a dsRNA-binding protein that includes two dsRBDs and a C4 domain (37). The two dsRBDs together express a much higher affinity for binding dsRNA than either one alone, confirming that the two domains cooperate for dsRNA binding (38, 39). However, a KR-helix motif in dsRBD2 gives it a stronger dsRNA-binding efficacy than dsRBD1 has (38). The C-terminal domain in *TRBP* binds to the tumor suppressor *Merlin*, the RNase III *Dicer*, and PKR activator (*PACT*) to create the *Medipal* domain (40). The C4 domain has a major influence on the reactions of *TRBP–PACT* and *TRBP–Dicer*.

**Figure 1:**
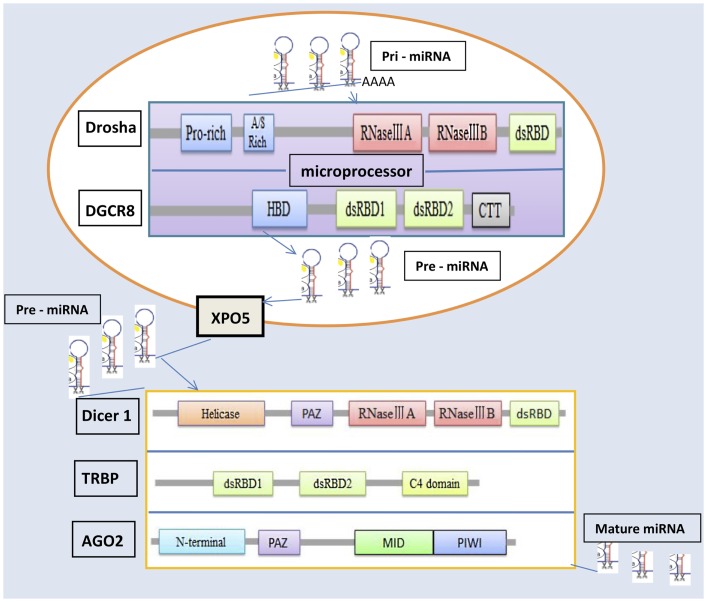
**Structure of microRNA machinery genes and miRNA biosynthesis**.

Figure 1 clarifies the molecular mechanisms underlying the miRNA processing machinery and the three-dimensional structures of the relevant proteins. Two RNase III domains, IIIA and IIIB are, common to *Drosha* and *Dicer1*. The *PAZ* domain is common to *Dicer1* and *AGO2* (23).

## Functions of miRNA Machinery Genes

Because of their core functions in miRNA processing, the genes *Drosha*, *DGCR8*, *Dicer1*, *XPO5*, *AGO2*, and *TRBP* are important in several aspects. *Drosha* can recognize and cleave the stem-loop structures in mRNAs, leading to their dysfunction, which occurs mostly in stem or progenitor cell populations (41–44). Due to the fact that the transcription factor Neurogenin 2 has a conserved hairpin-like pri-miRNAs do, *Drosha* can regulate the expression of this transcription factor (41). *Drosha* knockout mice are infertile due to oligoteratozoospermia or azoospermia, which suggests that *Drosha*-mediated miRNA production is important in male fertility (45). *Drosha* can affect the proliferation of human mesenchymal stem cells by regulating rRNA processing (46). Inhibition of *Drosha* also affects rRNA processing in HeLa cells (47).

*DGCR8* is also a part of a microprocessor, the *Drosha–DGCR8* complex, with an important function in miRNA maturation. This complex cleaves the hairpin structures in *DGCR8* mRNA (12). Deficiency of *DGCR8* results in altered short-term plasticity in the prefrontal cortex, affects dendritic spines and complexity, and alters brain miRNA biogenesis (48, 49). Moreover, inactivation of *DGRC8* in cardiac neural crest cells results in malformations and increased apoptosis (50). In addition, the loss of *DGCR8* in vascular smooth muscle cells results in liver hemorrhage, dilated blood vessels, and disarrayed vascular architecture in murine models, implying that the *DGCR8* gene plays an important role in vascular development by regulating the apoptosis and differentiation of these cells (51).

As an RNase III endonuclease, *Dicer* is a core enzyme which cleaves pre-miRNAs into 21- to 25-nt species in miRNA processing. *Dicer* has many important roles in the morphogenesis of developing tissues. For example, it plays an essential role in neuron polarity and neuronal development (52) and represses neuronal genes during endocrine cell maturation (53). Loss of *Dicer* results in gross abnormalities in cell number and function in the cortex and hippocampus (54), and deletion of *Dicer* in the early pancreatic lineage in *Pdx1–Cre* mouse models results in early pancreatic bud development and pancreas agenesis (55). On the other hand, *Dicer* is required for maintaining adult pancreas, and morphologic abnormalities in *Dicer1*-hypomorphic mice can be detected after 4 weeks of age (56). Deregulation of *Dicer1* in β-cells leads to progressive reduction in insulin secretion, glucose tolerance, and development of diabetes and impaired islet architecture (57, 58). Moreover, loss of *Dicer* results in significant reductions of testis mass and sperm number in germ cell knockout mouse as well as impaired meiotic progression (59). Finally, inactivation of *Dicer* in developing mouse lymphocytes can impair cell proliferation and survival and alter the repertoires of antigen receptors (60).

Mature RISC consists of a single-stranded small RNA bound to an *AGO* protein. *AGO* proteins can bind small interfering RNAs as well as miRNAs and mediate the repression of specific target RNAs either by degrading RNA or by inhibiting translation. Members of the *AGO* protein family have been implicated in both transcriptional and post-transcriptional gene silencing (31, 61). As a highly specialized member of the *AGO* family, *AGO2* has an essential non-redundant Slicer-independent function within the mammalian miRNA pathway. *AGO2* is also a key regulator of B lymphoid and erythroid development and function. However, deficiency in *AGO2* impairs miRNA biogenesis from pre-miRNAs and reduces miRNA expression levels (62). On the other hand, *AGO* protein-associated small RNAs repress mitogen-induced transcripts, and stabilized and stored mature miRNAs can be activated to regulate the mitogenic responses (63). Interestingly, *AGO2* in dopamine 2 receptor-expressing neurons regulates cocaine addiction (64), and nuclear *AGO2* has been reported to regulate voltage-gated potassium channels in adipose tissue-derived stromal cells with crucial functions in the self-renewal and cell de-aging processes (65). Furthermore, the interaction between the epidermal growth factor receptor gene (*EGFR*), a novel upstream regulator of the RISC-loading complex, and *AGO2* increases under hypoxia stress, which leads to elevated *AGO2-Y393* phosphorylation and inhibition of the transition of pre-miRNAs into mature miRNAs (66).

As a steroid receptor RNA activator-binding nuclear receptor coregulator, *TRBP* targets steroid-responsive promoters and regulates nuclear receptor activity and downstream gene expression (67). *TRBP* contributes to *HIV-1* gene expression by inhibiting the activation of the dsRNA-dependent protein kinase R (*PKR*) (68). Knockdown of *TRBP* can reduce the accumulation of hepatitis C virus RNA (69) and *TRBP* has been proposed as a target for antiviral therapies (68, 70, 71). The structures of *TRBP* and the *PKR* activator (*PACT*) are highly homologous (72). *TRBP* can control the *PACT* activation of *PKR* and the expression of the *HIV-1* gene (73). The interaction between *TRBP* and *PACT* may influence other cellular processes as well. *TRBP* can bind to the small-molecule enoxacin and express tumor suppressors in human cell cultures and mouse cancer models (74).

*XPO5* protein directly binds and mediates the nuclear export of dsRNA, including pre-miRNAs, viral hairpin RNAs, and tRNAs (16, 75). Inhibition of *XPO5* results in down-regulation of *Dicer* (76), global miRNA elevation disorder, and delayed G1/S transition (77), indicating that *XPO5* is a critical component in miRNA biogenesis, regulates global miRNA expression, and is associated with cell-cycle control. Because aberrant expression of *XPO5* increases the risk of cancer (78), it is a potential target for drug intervention.

## miRNA Machinery Genes in Cancer

Alterations in the miRNA machinery play important roles in the carcinogenesis of a variety of tumors (79). Preliminary analysis of data from The Cancer Genome Atlas consortium of multiple types of cancer through cBioPortal (80, 81) has shown a significant incidence of alterations in miRNA machinery genes (Table 1), especially the *AGO2* gene, which has a high incidence of gene alterations across cancer types, including breast invasive carcinoma (23.30%), colon and rectum adenocarcinoma (12.3%), bladder urothelial carcinoma (20.8%), and prostate adenocarcinoma (20.7%). This evidence supports prior reports linking miRNA-related alterations to cancers. The incidence of alterations mutation, copy number variation, and/or deregulated mRNA expression for these cancer types was 80.6, 95.4, 96.0, and 80.5%, respectively.

**Table 1:** **The Cancer Genome Atlas consortium data on the incidence of genetic alterations^a^ in microRNA machinery genes^b^ and driver genes^c^, by cancer type**.

Gene symbol	Breast invasive carcinoma (*n* = 463)		Colon and rectum adenocarcinoma (*n* = 195)		Bladder urothelial carcinoma (*n* = 125)		Prostate adenocarcinoma (*n* = 82)	
	*n*	%	*n*	%	*n*	%	*n*	%
***AGO2***	**108**	**23.3**	**24**	**12.3**	**26**	**20.8**	**17**	**20.7**
*APC*	40	8.6	153	78.5	10	8	6	7.3
*CCND1*	99	21.4	9	4.6	21	17	0	0
*CCNE1*	42	9.1	11	5.6	29	23.2	8	9.8
*CDKN2A*	61	13.2	14	7.2	56	44.8	6	7.3
*CHD1*	30	6.5	15	7.7	12	9.6	11	13.4
*CTCF*	41	8.9	17	8.7	11	8.8	7	8.5
*DEFB135*	15	3.2	6	3.1	12	9.6	8	9.8
***DGCR8***	23	5	9	4.6	**13**	**10.4**	2	2.4
***Dicer1***	**32**	**6.9**	**14**	**7.2**	15	12	3	3.7
*DNASE1*	31	6.7	6	3	8	6.4	4	4.9
***Drosha***	9	1.9	**29**	**14.9**	**42**	**33.6**	**6**	**7.3**
*EGFR*	30	6.5	23	11.8	25	20	4	4.9
*ERCC3*	33	7.1	20	10	18	14.4	8	9.8
*FRG1*	41	8.9	10	5	6	4.8	9	11
*GATA3*	96	20.7	13	6.7	21	16.8	4	4.9
*HIST1H2BA*	24	5.2	5	2.6	6	4.8	2	2.4
*KMT2C*	64	13.8	22	11.3	33	26.4	11	13.4
*KRAS*	33	7.1	84	43.1	15	12	10	12.2
*KRTAP6-2*	1	0.2	3	1.5	4	3.2	0	0
*LURAP1L*	6	1.3	14	7.2	9	7.2	13	15.9
*MAP3K1*	65	14	10	5.1	14	11.2	7	8.5
*MUC4*	40	8.6	12	6.2	19	15	20	24.4
*NKX3-1*	12	2.6	2	1	19	15	20	24.4
*OR2M4*	26	5.6	9	4.6	6	4.8	6	7
*OR51V1*	6	1.3	7	3.6	1	0.8	1	1.2
*OR6N1*	56	12.1	9	4.6	16	12.8	4	4.9
*PIK3R1*	36	7.8	16	8.2	7	5.6	11	13.4
*PPARG*	20	4.3	9	4.6	26	20.8	6	7.3
*PTEN*	54	11.7	20	10.3	13	10.4	30	36.6
*SOX4*	40	8.6	9	4.6	31	24.8	7	8.5
*RB1*	51	11	20	10.3	32	25.6	17	20.7
*TERC*	11	2.4	14	7.2	8	6.4	9	11
*TP53*	186	40.2	108	55.4	70	56	18	22
*TPX2*	25	5.4	71	36.4	24	19.2	10	12.2
***TRBP***	**40**	**8.6**	**16**	**8.2**	**9**	**7.2**	1	1.2
***XPO5***	**46**	**9.9**	**21**	**10.8**	**20**	**16**	**7**	**8.5**
*ZNF285*	4	0.9	7	4	18	14.4	6	7.3

*^a^Genetic alterations comprise of mutations and/or CNV and/or mRNA expression deregulation*.

*^b^miRNA machinery genes are in bold font*.

*^c^Driver genes are those with the highest incidence of alterations in a given dataset*.

Since the incidence of alteration of the *AGO2* gene was highest in breast invasive carcinoma, we analyzed the miRNA machinery genes in breast invasive carcinoma datasets to identify patterns of mutual genetic alterations and driver genes. A strong tendency of mutual exclusivity was noted for genetic alterations in the miRNA machinery gene *TRBP* with the driver genes *PIK3R1* (*p* = 0.03) and *KMT2C* (*p* = 0.0019) (Table 2). We also noted several incidences of co-occurrences in Table 2. Our analysis suggested that alterations in miRNA machinery genes interact with driver genes in at least a subset of tumors. Considering the regulatory role of miRNAs, the underlying mechanisms and cellular consequences of these interactions may be critical for understanding cancer pathology.

**Table 2:** **Patterns of mutual exclusivity and co-occurrence of driver genes and microRNA machinery gene alteration in The Cancer Genome Atlas consortium data for the breast invasive carcinoma dataset (*N* = 463)**.

Occurrence pattern	miRNA machinery gene	Driver gene^a^	*p* Value
**MUTUALLY EXCLUSIVE OCCURRENCE**			
	*AGO2*	*CTCF*	0.005
	*TRBP*	*KMT2C*	0.0019*
		*PIK3R1*	0.03*
**CO-OCCURRENCE**			
	*AGO2*	*PTEN*	0.0047
		*TP53*	0.00
	*XPO5*	*TP53*	0.0002
		*GATA3*	0.0001
	*DICER1*	*MAP3K1*	0.007
		*CTCF*	0.01

^a^Driver genes are those with the highest incidence of alterations in a given dataset. *Strong tendency toward mutual exclusivity 0 < odds ratio < 0.1. TCGA data obtained through cBioPortal (80, 81).

## miRNA Machinery Genes as Biomarkers for Cancers

Although the mechanism of microprocessor activity has been intensively investigated and dysregulation of miRNA machinery genes plays a pivotal role in the initiation and progression of malignancies, it remains largely unknown how miRNA machinery genes are regulated and whether they can serve as biomarkers for cancers. Abnormal expression of miRNA machinery genes has been found in a variety of human tumors (Table 3). The expression levels of *Drosha, DGCR8, Dicer, XPO5, AGO2*, and *TRBP* have all been associated with several cancers.

**Table 3:** **Expression levels of microRNA machinery genes in human tumors**.

miRNA machinery gene	Alteration type	Cancer type (reference)
*Drosha*	Up-regulation	BCC (82), SCC (82, 83), smooth muscle neoplasm (84)
	Down-regulation	Ovarian cancer (85), neuroblastoma (86), endometrial cancer (87), NPC (88), breast cancer (89), gallbladder adenocarcinoma (90)
*DGCR8*	Up-regulation	BCC (91), SCC (91), CRC (92), gastrointestinal cancer (93), ovarian cancer (94)
*XPO5*	Up-regulation	Urothelial carcinoma (95), breast cancer (96)
	Mutant	Non-small-cell lung cancer (19), renal cell carcinoma (79), CRC (97, 98), multiple myeloma (99)
*Dicer*	Up-regulation	SCC (67), prostate cancer (100), smooth muscle neoplasm (84)
	Down-regulation	Neuroblastoma (86), breast cancer (101), endometrial cancer (87), NPC (88), transitional cell carcinoma (102), gallbladder adenocarcinoma (90)
*AGO2*	Up-regulation	Prostate cancer (100), epithelial skin cancer (91), GC (103), hepatocellular carcinoma (104)
	Down-regulation	Lung adenocarcinoma (105), melanoma (106)
	Mutant	GC (97), CRC (97), breast cancer (107)
*TRBP*	Up-regulation	Prostate cancer (100), diffuse large B-cell lymphoma (108), adrenocortical carcinoma (109)
	Mutant	CRC cells (105), endometrial cancer cells (105)

*BCC, basal cell carcinoma; GC, gastric cancer; NPC, nasopharyngeal carcinoma; SCC, squamous cell carcinoma*.

The expression level of *Drosha* is up-regulated in basal cell carcinoma and squamous cell carcinoma (SCC) (82, 83), and elevated levels of *Drosha* are observed in smooth muscle neoplasms compared with smooth muscle, indicating that this enzyme is involved in smooth muscle neoplasms (85). Down-regulation of *Drosha* is associated with patient outcome in ovarian cancer (85), outcomes and risk groups in neuroblastoma (86), occurs in endometrial cancer (87), correlates with nasopharyngeal carcinoma and the patient outcomes (88), is associated with the specific subgroups of breast cancer (89), and is associated with metastasis, invasion, and poor prognosis in gallbladder adenocarcinoma (90).

*DGCR8* expression levels are over-expressed in basal cell carcinoma (110), SCC (110), colorectal cancer (CRC) (91), gastrointestinal cancer (92), and ovarian cancer (93). Knockdown of *DGCR8* in ovarian cancer cells disturbs their proliferation, migration, and invasion and increases their sensitivity to the chemotherapeutic drug cisplatin (93), which suggests that an elevated level of *DGCR8* is associated with carcinogenesis.

*Dicer* is down-regulated in many tumors, such as transitional cell carcinoma of the urinary bladder (94), neuroblastoma (86), nasopharyngeal carcinoma (88), endometrial cancer (87), breast cancer (102), lung cancer (101, 111), gastric cancer (GC) (112), ovarian cancer (113), and gallbladder adenocarcinoma (90). Repression of *Dicer* is associated with poor prognosis for patients with lung cancer (101), ovarian cancer (114), chronic lymphocytic leukemia (115), or colorectal CRC (116), and it promotes cell proliferation in A2780 and SKOV3 ovarian cancer cells (117). Conversely, compared with normal tissue, the expression of *Dicer* is higher in cutaneous SCC (82), salivary gland pleomorphic adenoma (118), acute myeloid leukemia (119), smooth muscle neoplasm (85), and prostate cancer (100). Overexpression of *Dicer* has been shown to lead to poor survival in patients with soft tissue sarcoma (84). Loss of *Dicer* expression suppresses the growth and oncogenicity of human prostate cancer cell lines but enhances migratory capacity in some prostate cancer cell lines (120). *Dicer* is increased in human prostate cancer specimens, but lower Dicer expression predicts faster cancer recurrence (120). Complete ablation and hemizygous loss of *Dicer* reduced tumor growth. Hemizygous loss also resulted in an invasive phenotype and causes seminal vesicle obstruction, which indicated that the regulation of *Dicer* depends on dosage and context (120).

The expression of *AGO2* is up-regulated in GC (103), epithelial skin cancer (110), prostate cancer (100), and hepatocellular carcinoma (104). *AGO2* binds to the tumor metastasis factor focal adhesion kinase promoter and triggers its transcription, which suggests a new function of *AGO2* in tumor progression (104). Repression of *AGO2* protein has been found in human lung adenocarcinomas (105) and in melanoma, for which the mRNA level of *AGO2* did not change (106). Overexpression of *AGO2* has been shown to inhibit cancer cell proliferation and migration in mice models (105). The stability of *AGO2* protein is essential, as is the frame shift mutation of the *AGO2* gene in GC and CRC with high microsatellite instability (MSI-H), which suggests that these alterations are risk factors for GC and CRC (97). Single-nucleotide polymorphisms of *AGO2* have been associated with the outcome of breast cancer patients (107).

Compared with in lymph nodes, *TRBP* is over-expressed in prostate cancer (116). Similarly, *TRBP* is over-expressed in diffuse large B-cell lymphoma and is associated with a poor chemotherapy response (108). Both *TRBP* mRNA and *TRBP* protein levels are higher in adrenocortical carcinomas than in adenomas or adrenal cortices (109). Knockdown of *TRBP* decreases cell proliferation and induces cell apoptosis in diffuse large B-cell lymphoma cells (108) and adrenocortical carcinomas cells (109). However, the expression levels of *TRBP* are not significantly different between patients with epithelial skin cancer and persons who do not (110). Melo et al. found that the presence of inactivating mutations in *TRBP* gene in human cancer cell lines and primary tumors with MSI-H impaired miRNA processing and enhanced cellular transformation and the loss of *TRBP* led to a secondary defect in *Dicer1* activity. These results further confirmed the role of loss of function events in the regulation of miRNA processing machinery during tumorigenesis (121).

Dysfunction of *XPO5* can also result in carcinogenesis. The expression level of *XPO5* is up-regulated in urothelial carcinoma of the bladder (95) and breast cancer (96) and is positively correlated with tumor development and invasion (95). The *XPO5* mutant *rs11077* increases the risk of renal cell carcinoma (79), is associated with chemotherapy response and survival of patients with advanced non-small-cell lung cancer (24), and is associated with the outcomes of patients with multiple myeloma undergoing autologous stem cell transplantation (99). The discoveries of a mutation in a CRC patient (97) and two CRC cell lines, HCT-15 and DLD-1 (98), with MSI-H imply that the *XPO5*-inactivating mutant results in pre-miRNA accumulating in the nucleus. The restoration of *XPO5* repairs the impaired export and expresses tumor suppressor features (98).

Additional analysis of the expression levels of these miRNA machinery genes and alterations and their interactions with their driver genes in tumors could discriminate cancer patients from healthy controls and be associated with the outcomes of cancer patients.

## Future Perspectives

Along with conducting intensive studies of tumor-associated miRNAs and miRNA machinery genes, which play crucial roles in tumorigenesis, scientists are focusing on the miRNA machinery genes *Drosha*, *DGCR8*, *XPO5*, *Dicer*, *AGO2*, and *TRBP* for their potential as cancer biomarkers. The mechanisms involved in miRNA maturation still need to be explored, and new functions of some known genes in miRNA maturation need be uncovered, such as the *EGFR* gene was induced miRNAs mature as a regulator of *AGO2* (71) and *ADAR1* formed a complex with *Dicer* through direct interaction and regulated miRNA processing (122). The dysregulation of miRNA machinery genes (mutation, up-regulation, or down-regulation) can result in oncogenicity and poor patient outcomes. The functions of miRNA machinery genes will be difficult to comprehend because the same gene can have different functions in different types of cancers, and these functions may be not only dosage-dependent but also tissue-dependent (118). Finally, scientists need to explore the different roles of miRNA machinery genes in the physiology and pathology of tumorigenesis. Understanding these roles will help us to use miRNA to develop cancer biomarkers, experimental tools, and antitumor therapy.

## Conflict of Interest Statement

The authors declare that the research was conducted in the absence of any commercial or financial relationships that could be construed as a potential conflict of interest.

## Abbreviations

AGO2, argonaute 2; CRC, colorectal cancer; DGCR8, diGeorge critical region 8; dsRBD, double-stranded RNA-binding domain; dsRNA, double-stranded RNA; GC, gastric cancer; miRNA, microRNA; MSI-H, high microsatellite instability; PACT, PKR activator; PKR, protein kinase R; pre-miRNA, precursor miRNA; pri-miRNA, primary miRNA; RISC, RNA-induced silencing complex; RNase III, ribonuclease III; SCC, squamous cell carcinoma; TRBP, TAR RNA-binding protein; XPO5, exportin-5.
